# Unmet Psychosocial Needs of Health Care Professionals in Europe During the COVID-19 Pandemic: Mixed Methods Approach

**DOI:** 10.2196/45664

**Published:** 2023-09-06

**Authors:** Svenja Hummel, Ina Michelsen, Ali Zafar, Steffen Moritz, Charles Benoy, Cédric Lemogne, Rosa Almeida, Raquel Losada, Oscar Ribeiro, Vincenza Frisardi, Ilaria Tarricone, Silvia Ferrari, Frieder Dechent, Christian G Huber, Steffi Weidt, Gwendolyn Mayer, Jobst-Hendrik Schultz

**Affiliations:** 1 Department of General Internal Medicine and Psychosomatics Heidelberg University Hospital Heidelberg Germany; 2 Department of Psychiatry and Psychotherapy University Medical Center Hamburg-Eppendorf Hamburg Germany; 3 Centre Hospitalier Neuro-Psychiatrique Luxembourg Ettelbrück Luxembourg; 4 Université Paris Cité and Université Sorbonne Paris Nord, Inserm, INRAE, Center for Research in Epidemiology and StatisticS (CRESS), Service de Psychiatrie de l’adulte AP-HP, Hôpital Hôtel-Dieu Paris France; 5 INTRAS Research, Innovation and Knowledge Unit, Intras Foundation Valladolid Spain; 6 Center for Health Technology and Services Research (CINTESIS) at the Associate Laboratory RISE - Health Research Network Department of Education and Psychology University of Aveiro Aveiro Portugal; 7 Geriatric Unit, IRCCS-AOU BO, Policlinico San't Orsola Bologna Italy; 8 Department of Medical and Surgical Sciences Bologna University Bologna Italy; 9 Department of Biomedical, Metabolic and Neural Sciences University of Modena & Reggio Emilia Modena Italy; 10 University Psychiatric Clinics Basel University of Basel Basel Switzerland; 11 Department of Psychiatry, Psychotherapy and Psychosomatics, Psychiatric University Hospital Zurich and University of Zurich Zurich Switzerland

**Keywords:** COVID-19, mental health, health care professionals, health care workers, pandemic preparedness, mixed methods, coping, stressors, psychosocial

## Abstract

**Background:**

The COVID-19 pandemic severely affected everyday life and working conditions for most Europeans, particularly health care professionals (HCPs). Over the past 3 years, various policies have been implemented in various European countries. Studies have reported on the worsening of mental health, work-related stress, and helpful coping strategies. However, having a closer look is still necessary to gain more information on the psychosocial stressors and unmet needs of HCPs as well as nonmedical staff.

**Objective:**

This study aimed to obtain quantitative information on job-related stressors of physicians and nurses and the coping strategies of HCPs and nonmedical staff at 2 periods of the COVID-19 pandemic. By further analyzing qualitative comments, we wanted to gain more information on the psychosocial stressors and unmet needs of HCPs as well as nonmedical staff on different levels of experience.

**Methods:**

A cross-sectional survey was conducted at 2 time points during the COVID-19 pandemic in several European countries. The first study period (T1) lasted between April 1 and June 20, 2020, and the second study period (T2) lasted between November 25, 2021, and February 28, 2022. On a quantitative level, we used a questionnaire on stressors for physicians and nurses and a questionnaire on coping strategies for HCPs and nonmedical staff. Quantitative data were descriptively analyzed for mean values and differences in stressors and coping strategies. Qualitative data of free-text boxes of HCPs and nonmedical staff were analyzed via thematic analysis to explore the experiences of the individuals.

**Results:**

T1 comprised 609 participants, and T2 comprised 1398 participants. Overall, 296 participants made 438 qualitative comments. The uncertainty about when the pandemic would be controlled (T1: mean 2.28, SD 0.85; T2: mean 2.08, SD 0.90) and the fear of infecting the family (T1: mean 2.26, SD 0.98; T2: mean 2.02, SD 1.02) were the most severe stressors identified by physicians and nurses in both periods. Overall, the use of protective measures (T1: mean 2.66, SD 0.60; T2: mean 2.66, SD 0.60) and acquiring information about COVID-19 (T1: mean 2.29, SD 0.82; T2: mean 1.99, SD 0.89) were identified as the most common coping strategies for the entire study population. Using thematic analysis, we identified 8 themes of personal experiences on the micro, meso, and macro levels. Measures, working conditions, feelings and emotions, and social climate were frequently mentioned topics of the participants. In T1, feelings of isolation and uncertainty were prominent. In T2, feelings of exhaustion were expressed and vaccination was frequently discussed. Moreover, unmet psychosocial needs were identified.

**Conclusions:**

There is a need for improvement in pandemic preparedness. Targeted vocational education measures and setting up of web-based mental health support could be useful to bridge gaps in psychosocial support needs in future crises.

## Introduction

### Background

The COVID-19 pandemic had a drastic impact on daily life and working conditions in Europe. By mid-March 2020, preventive measures such as physical distancing were implemented across European countries such as France, Italy, Spain, the United Kingdom, and Belgium. In many countries, a shortage of health care resources required reasonable distribution and sustainable use of personal protective equipment (PPE), human resources, and intensive care unit (ICU) beds. Triaging patients with COVID-19 became necessary. The collapse of the health care system proved serious in some countries such as Italy. Spain was more affected than neighboring countries, such as Portugal, which had fewer reported cases in March 2020. Before COVID-19, there were 9.7 ICU beds per 100,000 people in Spain versus 33.9 ICU beds per 100,000 people in Germany [1]. At the beginning of the pandemic, Austria, Germany, and Switzerland were successful in managing the crisis and reported lower mortality rates than Belgium, the United Kingdom, France, Spain, and Italy. Early efforts were made to contain the number of cases, such as the implementation of information websites. In Germany, for example, health care system preparedness was improved by rapidly creating new ICU resources rather than repurposing them, as was the case in Switzerland and Austria. Furthermore, there was a large increase in video consultations in terms of telehealth. However, in some cases, policies in different federal states within the country were not consistent [2].

As virus transmission subsided, eventually, these policies were eased [3]. From June to September 2020, borders were reopened [4] and detailed testing protocols were established for health care professionals (HCPs) [5]. Italy was one of the first countries to mandate vaccinations for HCPs in May 2021. By September 2021, mandatory vaccination protocols for HCPs were adopted by Greece and France [6], followed by Germany [7]. Austria became the first country to implement mandatory vaccinations for all people. Italy and Greece followed the mandatory vaccination for people above a certain age [8]. In some European countries such as Germany, France, and Italy, only completely vaccinated individuals or those who had already been infected with COVID-19 had access to particular public spaces [7].

Emergency measures such as curfews, school closures, and a shift to working from home or job losses are a few examples of the challenges that people had to face. With the introduction of vaccines, the topic of vaccine hesitancy became a focus of attention [9]. As many new policies were introduced during the COVID-19 pandemic, it was not surprising that the general population in Europe was distressed and, in many cases, had mental health problems [10]. On the one hand, a COVID-19 infection directly impacted mental health [11], whereas on the other hand, there was the deterioration of mental health among the general population [12] as an indirect effect of the pandemic, for example, in terms of psychosocial problems. A psychosocial problem can originate in a variety of domains (eg, environmental problems, occupational problems, inadequate social support, educational problems, inadequate access to health and other services, and interpersonal losses), which can have a negative influence on the individual and might cause mental illnesses [13].

The COVID-19 pandemic posed major challenges for health care systems and HCPs worldwide.

For European HCPs, there was an increase in workload [14]. In the nursing profession, staff shortages and increased workloads were already commonplace before the pandemic [15] and, during COVID-19, the burnout of nurses was identified [16]. The negative impact of mental health on HCPs due to COVID-19 has already been proven [17,18], whereby nurses and frontline HCPs were particularly strained psychologically. Nevertheless, in a study by Hummel et al [19], we found significantly lower mean scores for depression and anxiety among HCPs than among nonmedical staff.

As nurses and frontline HCPs (eg, physicians) seem to be the most affected groups during the pandemic, it is worth focusing on these professions regarding work-related stressful events (hereafter referred to as stressors and psychosocial burden). Stress occurs when the demands overwhelm the person [20]. Several stressors of HCPs were identified during the severe acute respiratory syndrome (SARS) outbreak in 2003 [21] and during the COVID-19 pandemic [22]. In 1 study, nurses rated the fear of infection as the most important stressor, followed by death [23]. Coping can be understood as an adaptation that helps the individual deal with challenges that exceed their capacities [20]. Coping strategies are, in our context, to be understood as profession-independent ways of dealing with the strain of the COVID-19 pandemic in general. Taking personal protective measures was a frequently used coping strategy by nurses and physicians along with talking to family and friends or doing relaxation activities in a study on the COVID-19 pandemic by Rose et al [22].

By adding qualitative data, one might gain more insights into the participants’ way of thinking than by using only the results of quantitative data. Responses to such questions can differ in length and detail, and the participants have the opportunity to explain themselves in detail. Open-ended survey questions help to supplement the quantitative results by providing additional information [24], which can be helpful in exploring psychosocial factors and unmet needs. According to social work research, individuals can be studied at a macro, meso, and micro level. The micro level describes the level of the individual, the meso level describes the interaction between groups, and the macro level addresses social structures and institutions [25]. Considering the deterioration of HCPs and the nonmedical staff’s mental health during the COVID-19 pandemic, assessments are needed with different levels of individual experiences (macro, meso, and micro) to identify existing gaps in psychosocial care.

### Objectives

This study aimed to gain quantitative information on work-related stressors of physicians and nurses and the coping strategies of HCPs and nonmedical staff during 2 periods of the COVID-19 pandemic. By further analyzing the qualitative comments, we obtained more information on the psychosocial stressors and unmet needs of HCPs as well as nonmedical staff at different levels of experience.

## Methods

### Study Design

A cross-sectional web-based survey was conducted at 2 different stages during the COVID-19 pandemic. The first period lasted from April 1 to June 20, 2020, during the first lockdown in Europe [26]. The second phase lasted between November 25, 2021, and February 28, 2022, when the second and third lockdowns ended in Europe [27,28].

### Ethics Approval

The study was approved by the ethics committee of the Heidelberg University Medical Faculty (S-361/2020).

### Data Collection, Informed Consent, and Participation

Data collection was conducted in compliance with the European General Data Protection Regulation. The survey questionnaire was distributed in 10 European countries, and all health care workers and associated staff at hospitals as well as nonmedical staff were eligible to participate. Consent to participate was included in the web-based questionnaire and obtained without a signature. No allowance was provided for participation in the survey. All the questionnaires were completed anonymously. Data security was granted using the Secure Sockets Layer–encrypted platform, SoSci Survey [29].

### Questionnaire

A questionnaire comprising 3 sections on demographics, stress factors, and coping strategies was administered. As no COVID-19–specific questionnaires were available at the beginning of the pandemic, we derived our instrument from a study by Lee et al [21] on SARS. The original 23-item questionnaire focused on work-related stressors for nurses during SARS. Therefore, we modified the questionnaire to include physicians and nurses working in hospitals [19]. For the stress factors section, participants indicated on a Likert scale from 0 (not at all) to 4 (very much) how often they thought about or were concerned about 23 specific stressors in their everyday lives or at clinical work. Only physicians and nurses were eligible to complete this section.

The section on coping strategies was also derived from the study by Lee et al [21]. Participants responded to 12 items on a scale from 0 (almost never) to 3 (almost always) to assess how frequently these 12 coping strategies were applied in their everyday lives. This section was available to all participants, as it did not focus on medical issues. Considering the utility of gaining qualitative data, at the end of the questionnaire, we included an open-ended question with free-text boxes for the participants to leave one or more comments at the end of the survey. To ensure neutral responses within the context of the study objectives, the question was framed as, “If there is anything else you would like to tell us, you can do so here.”

### Recruitment

The questionnaire was translated by native speakers or professional translators into several European languages, including English, Italian, German, French, Spanish, and Portuguese, to obtain a larger sample population. Accordingly, the questionnaire was also distributed in the respective countries. It was made available on the web via the platform SoSci Survey [29]. The link to the survey was distributed via email to our personal and professional networks using the snowball sampling method. Invitation emails were sent to colleagues at the Heidelberg University Hospital and further distributed to related institutions and to European contacts with their partner organizations, hospitals, and professional associations. Participants were also recruited via personal networks or public social networking groups, such as Twitter, LinkedIn, and Facebook.

### Participants

The HCPs included physicians and nurses, as well as a group of *other job in health care* comprising health care psychologists, physiotherapists, other nursing professions, laboratorians, technicians, and occupational therapists. The nonmedical staff was a heterogeneous group, which, for example, consisted of administrators, managers, engineers, teachers, retired persons, or secretaries. However, not all nonmedical participants revealed their actual profession.

### Data Analysis

#### Overview

Quantitative and qualitative data were analyzed independently. In the first study period, the sample comprised 609 participants, of whom 78 submitted 111 qualitative comments. In the second phase, 1398 participants completed the survey, with 218 participants submitting 327 qualitative comments. Overall, 438 qualitative comments were made by 296 participants, of whom 31.4% (n=93) were nurses, 17.2% (n=51) were physicians, and 19.9% (n=59) were nonmedical staff. As the stressors’ questionnaire was specific for people working at hospitals, we only analyzed the answers from physicians and nurses for this questionnaire. The coping questionnaire was analyzed for the entire study population. For not fulfilling the inclusion criteria, 69 cases were excluded from the data analysis of the first phase, whereas 41 cases were excluded from the second period. The population demographics and quantitative results for the first study period were published earlier [19].

#### Quantitative Data

Missing data occurred only for the stressors section during the first study period. Questionnaires with >10% missing data per participant were excluded using a pragmatic and rigor approach following previous recommendations [30]. In total, we analyzed 346 questionnaires for stressors in the first period and 696 in the second period. For coping strategies, 609 questionnaires were analyzed in the first period and 1398 in the second period.

Means and SDs were calculated for all items on stressors for physicians and nurses and coping strategies for all participants. We used Cronbach α to determine the internal consistency of the stressors and coping scales for both study periods. Cronbach α >.80 was considered a threshold for acceptance [31,32]. For the stressors questionnaire, the internal consistency of the first study period was assessed from a Cronbach α of .92 and that of the second study period was assessed from a Cronbach α of .93. For the coping questionnaire, the internal consistency of the first study period was assessed from a Cronbach α of .74 and of the second study period was assessed from a Cronbach α of .72. *t* tests (2-tailed) were conducted for independent groups to compare the mean values of stressors and coping items of the 2 study periods. In all analyses, *P* values <.05 were considered statistically significant. Quantitative data were analyzed using SPSS (version 26; IBM Corp) [33].

#### Qualitative Data

All comments in the free-text comment boxes by HCPs and nonmedical staff were considered for analysis. We provided the demographics of our qualitative data with means and SDs for age, frequencies, and percentages. The length of the comments made by the participants ranged from 3 words (eg, “not enough staff”) to >100 words. The comments were translated and analyzed for each study period separately using inductive thematic analysis [34] to identify themes that tell a story about what concerns people beyond quantitative data. We used the 6-step approach developed by Braun and Clarke [35].

Codes and resulting themes were elaborated by 2 authors (SH and IM). After each step, the authors discussed the results to create a common theme map. Second-level and third-level themes were developed when the first-level theme revealed multiple aspects. The key themes identified were located at different levels in relation to the individual based on the levels of experience. Therefore, the themes were divided into *macro*, *meso*, and *micro* levels based on recommendations for social work research [25].

**Figure 1:**
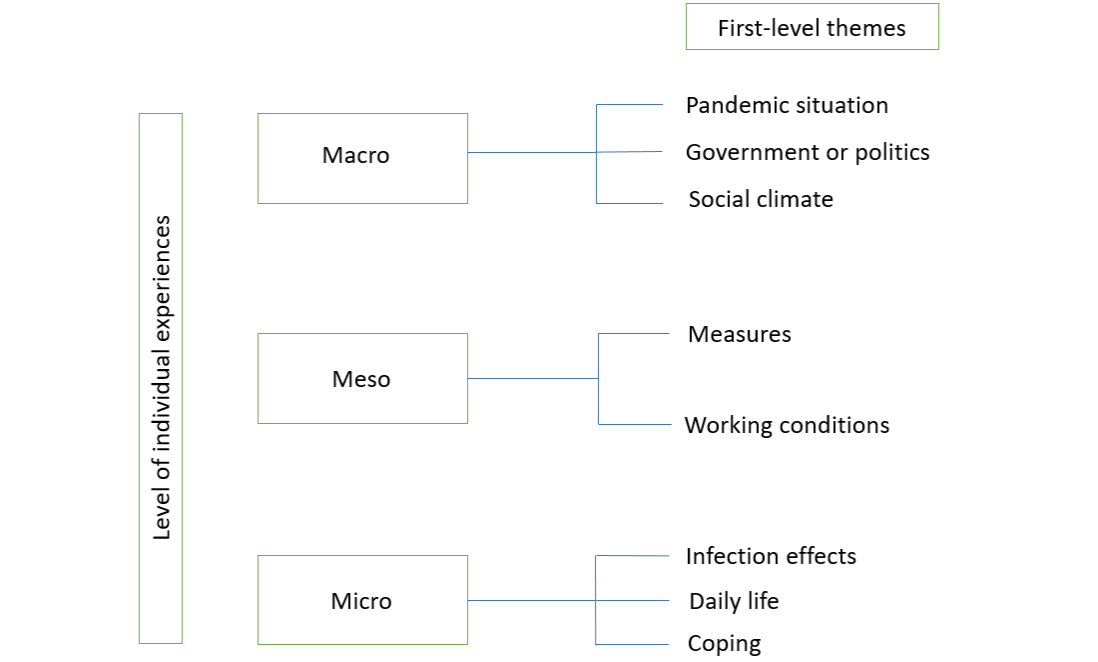
First-level themes at their assigned levels of individual experience.

As the *pandemic situation*, *government or politics*, and *social climate* themes described the overarching sociopolitical impacts of COVID-19, these themes were categorized to the macro level. The topics *measures* and *working conditions* could be located at a more individual impact level and were assigned the meso level. The micro level included topics dealing with the individual and included the themes *daily life*, *coping*, and *infection effects* (Figure 1).

The 8 key themes and the most important second- and third-level themes are presented in this paper, with relevant quotes to illustrate the themes. The complete list of themes is available in Multimedia Appendix 1 [36].

## Results

### Quantitative Data

#### Stressors for Physicians and Nurses

For both study periods, *Uncertainty about when the epidemic will be under control* was rated as the most stressful factor, followed by *Worry about inflicting COVID-19 on family*. In the first period, *Worry about nosocomial spread* and *Frequent modification of infection control procedures* were the third and fourth biggest stressors, respectively. In the second period, *Worry about lack of manpower* was the third and *Frequent modification of infection control procedures* was the fourth biggest stressor. The highest significant mean difference (t_752.58_=10.09, *P*<.001) between first and second study period was found for *Worry about nosocomial spread* (first study period: mean 2.04, SD 0.91; second study period: mean 1.41, SD 1.01; mean difference 0.63, 95% CI 0.50-0.75).

Another item with the most significant mean difference (t_656.97_=7.39, *P*<.001) was *Worry about lack of proper knowledge and equipment* (first study period: mean 1.66, SD 1.04; second study period: mean 1.17, SD 1.17; mean difference 0.50, 95% CI 0.36-0.63). The mean values and 2-tailed *t* test results for all the items are presented in Table 1.

**Table 1:** Stressors for nurses and physicians during COVID-19 for both study periods (first n=346 and second n=696).

Items^a^	First study period		Second study period		*t* test (*df*)	*P* value
	Values, n (%)	Values, mean (SD)	Values, n (%)	Values, mean (SD)		
Uncertainty about when the epidemic will be under control	345 (99.7)^b^	2.28 (0.85)^b^	696 (100)^b^	2.08 (0.90)^b^	3.48 (1039)	.001^c^
Worry about inflicting COVID-19 on family	346 (100)^b^	2.26 (0.98)^b^	696 (100)^b^	2.02 (1.02)^b^	3.60 (1040)	<.001^c^
Worry about nosocomial spread	345 (99.7)^b^	2.04 (0.91)^b^	696 (100)	1.41 (1.01)	10.09 (752.58)	<.001^c^
Frequent modification of infection control procedures	345 (99.7)^b^	2.03 (0.88)^b^	696 (100)^b^	1.73 (0.96)^b^	5.03 (741.61)	<.001^c^
Protective gears cause physical discomfort	345 (99.7)	1.75 (1.02)	696 (100)	1.50 (1.06)	3.57 (1039)	<.001^c^
Deterioration of patients’ condition	345 (99.7)	1.70 (1.00)	696 (100)	1.52 (1.04)	2.70 (1039)	.01^c^
Worry about lack of proper knowledge and equipment	346 (100)	1.66 (1.04)	696 (100)	1.17 (0.99)	7.39 (656.97)	<.001^c^
Worry about being negligent and endangering patients	346 (100)	1.66 (1.07)	696 (100)	1.25 (1.04)	6.03 (1040)	<.001^c^
Worry about getting infected	346 (100)	1.62 (1.03)	696 (100)	1.41 (1.00)	3.10 (1040)	.002^c^
Patients’ emotional reaction	346 (100)	1.57 (0.96)	696 (100)	1.53 (0.99)	0.61 (1040)	.54
Worry about lack of manpower	346 (100)	1.57 (1.05)	696 (100)^b^	1.97 (1.04)^b^	−5.86 (1040)	<.001^c^
Documentation and reporting procedures unclear	345 (99.7)	1.54 (1.01)	696 (100)	1.26 (1.00)	4.17 (1039)	<.001^c^
Patient families’ emotional reaction	345 (99.7)	1.52 (1.01)	696 (100)	1.44 (1.01)	1.26 (1039)	.21
Coworkers being emotionally unstable	346 (100)	1.53 (0.97)	696 (100)	1.59 (1.00)	−0.92 (1040)	.36
Being without properly fitted environment	346 (100)	1.51 (1.08)	696 (100)	1.27 (1.06)	3.47 (1040)	.001^c^
Conflict between duty and safety	346 (100)	1.48 (1.07)	696 (100)	1.30 (1.00)	2.66 (649.35)	.01^c^
Worry about being negligent and endangering coworkers	346 (100)	1.48 (1.04)	696 (100)	0.98 (0.97)	7.44 (652.43)	<.001^c^
Be infected by the colleagues	346 (100)	1.30 (1.01)	696 (100)	1.10 (0.97)	3.08 (662.88)	.002^c^
Protective gears being a drag in providing quality care	346 (100)	1.28 (1.05)	696 (100)	1.15 (1.04)	1.93 (1040)	.05
Coworkers displaying COVID-19–like symptoms	346 (100)	1.25 (0.97)	696 (100)	1.09 (0.96)	2.54 (1040)	.01^c^
Equivocal definition of the responsibility between physicians and nurses	346 (100)	1.19 (1.04)	696 (100)	1.08 (1.02)	1.57 (1040)	.12
Yourself displaying COVID-19-like symptoms	346 (100)	1.12 (1.04)	696 (100)	1.05 (0.98)	1.00 (1040)	.32
Blaming from commanding officers	345 (99.7)	0.70 (0.95)	696 (100)	0.85 (1.04)	−2.38 (1039)	.02^c^

^a^Mean and SD for the question: “When you think about COVID-19 in your life and work, how often did you think or worry about the following things?” (0=not at all and 3=very much).

^b^Frequently used stressor.

^c^*P* value <.05 was considered statistically significant.

#### Coping Strategies of the Whole Population

*Taking protective measures (washing hands, wearing mask, taking own temperature, etc)* was the most often used coping strategy, followed by *Actively acquiring more knowledge about COVID-19 (symptoms, transmission pathway, etc)* in both study periods. In the first period, the third most reported coping strategy was *Video-chatting with family and friends by phone to share concerns and support*, whereas in the second period, it was *Engaging in recreational activities (web-based shopping, social media, internet surfing...)*.

The highest significant mean difference (t_1218.57_=11.14, *P*<.001) between the first and second periods was found for *Video-chatting with family and friends by phone to share concerns and support* (first study period: mean 1.85, SD 0.88; second study period: mean 1.36, SD 0.93; mean difference 0.49, 95% CI 0.40-0.57). Another item with a significant mean difference (t_2005_=7.09, *P*<.001) was *Actively acquiring more knowledge about COVID-19 (symptoms, transmission pathway, etc*; first study period: mean 2.29, SD 0.82; second study period: mean 1.99, SD 0.89; mean difference 0.30, 95% CI 0.22-0.38). The mean values and 2-tailed *t* test results for all the items are presented in Table 2.

**Table 2:** Coping strategies during COVID-19 for the whole study population of both study periods (first n=609 and second n=1398).

Items^a^	First study period		Second study period		*t* test (*df*)	*P* value
	Values, n (%)	Values, mean (SD)	Values, n (%)	Values, mean (SD)		
Taking protective measures (washing hands, wearing mask, taking own temperature, etc)	609 (100)^b^	2.66 (0.60)^b^	1398 (100)^b^	2.66 (0.60)^b^	0.06 (2005)	.95
Actively acquiring more knowledge about COVID-19 (symptoms, transmission pathway, etc)	609 (100)^b^	2.29 (0.82)^b^	1398 (100)^b^	1.99 (0.89)^b^	7.09 (2005)	<.001^c^
Video-chatting with family and friends by phone to share concerns and support	609 (100)^b^	1.85 (0.88)^b^	1398 (100)	1.36 (0.93)	11.14 (1218.57)	<.001^c^
Engaging in recreational activities (web-based shopping, social media, internet surfing...)	609 (100)	1.67 (0.94)	1398 (100)^b^	1.57 (0.90)^b^	2.18 (2005)	.03^c^
Engaging in health-promoting behaviors (more rest, exercise, balanced diet, etc)	609 (100)	1.63 (0.97)	1398 (100)	1.41 (0.99)	4.57 (2005)	<.001^c^
Switching thoughts and facing the situations with positive attitude	609 (100)	1.58 (0.88)	1398 (100)	1.45 (0.92)	2.93 (2005)	.003^c^
Limiting oneself from watching too much news about COVID-19	609 (100)	1.42 (0.98)	1398 (100)	1.55 (0.99)	−2.71 (2005)	.01^c^
Distracting oneself from thinking about COVID-19 issues by suppression or keeping busy	609 (100)	1.35 (0.94)	1398 (100)	1.28 (0.94)	1.62 (2005)	.11
Acquiring mental health knowledge and information	609 (100)	1.10 (0.97)	1398 (100)	1.13 (0.97)	−0.63 (2005)	.53
Practicing relaxation methods (meditation, yoga, Tai Chi, etc)	609 (100)	0.57 (0.91)	1398 (100)	0.76 (0.97)	−4.04 (1226.25)	<.001^c^
Venting emotions by crying, screaming, smashing things, and so on	609 (100)	0.54 (0.85)	1398 (100)	0.44 (0.76)	2.48 (1065.30)	.01^c^
Using alcohol or drugs	609 (100)	0.35 (0.65)	1398 (100)	0.39 (0.66)	−1.45 (1203.59)	.15

^a^Mean and SD for the question “When you think about COVID-19 in your life and work. How often did you use or try to use the following methods to handle the situation?” (from 1=almost never to 4=almost ever) for all participants in the order of their frequency of use.

^b^Frequently used coping strategy.

^c^*P* value <.05 was considered statistically significant.

### Qualitative Results

#### Study Participants

Table 3 provides the sociodemographic characteristics of the study population for the qualitative analysis of each study period. For the overall population, the age range was 22 to 76 years.

**Table 3:** Demographic characteristics of the study participants in the first period, second period, and total.

Characteristics				First study period (n=78)		Second study period (n=218)		Total (n=296)
Age (years), mean (SD)				46.12 (12.74)		44.53 (11.09)		45.33 (11.92)
**Gender, n (%)**								
	Man		26 (33.3)		48 (22)		74 (25)	
	Woman		52 (66.7)		169 (77.5)		221 (74.7)	
	Nonbinary		0 (0)		1 (0.5)		1 (0.3)	
**Country, n (%)**								
	United Kingdom		11 (14.1)		0 (0)		11 (3.7)	
	Germany		18 (23.1)		35 (16.1)		53 (17.9)	
	Austria		5 (6.4)		0 (0)		5 (1.7)	
	Switzerland		5 (6.4)		25 (11.5)		30 (10.1)	
	France		10 (12.8)		76 (34.9)		86 (29.1)	
	Italy		12 (15.4)		7 (3.2)		19 (6.4)	
	Spain		13 (16.7)		4 (1.8)		17 (5.7)	
	Portugal		4 (5.1)		2 (0.9)		6 (2)	
	Belgium		0 (0)		16 (7.3)		16 (5.4)	
	Luxemburg		0 (0)		53 (24.3)		53 (17.9)	
**Language, n (%)**								
	English		12 (15.4)		1 (0.5)		13 (4.4)	
	German		27 (34.6)		84 (38.5)		111 (37.5)	
	Italian		12 (15.4)		7 (3.2)		19 (6.4)	
	Spanish		13 (16.7)		4 (1.8)		17 (5.7)	
	Portuguese		4 (5.1)		3 (1.4)		7 (2.4)	
	French		10 (12.8)		119 (54.6)		129 (43.6)	
**Profession, n (%)**								
	**Health care professional**							
		Physicians	20 (25.6)		31 (14.2)		51 (17.2)	
		Volunteers	1 (1.3)		0 (0)		1 (0.3)	
		Dentist	1 (1.3)		0 (0)		1 (0.3)	
		Nurse	17 (21.8)		76 (34.9)		93 (31.4)	
		Other job in health care	13 (16.7)		78 (35.8)		91 (30.7)	
	Nonmedical		26 (33.3)		33 (15.1)		59 (19.9)	

#### Themes

From the thematic analysis, 8 key themes emerged, namely *pandemic situation*, *government or politics*, *social climate*, *measures*, *working conditions*, *infection effects*, *daily life*, and *coping*. Among these key themes, 27 second-level themes were identified. For some second-level themes, third-level themes were also identified. A total of 3 themes were assigned to the macro level of experience, 2 referred to the meso level, and 3 referred to the micro level of experience. Most topics were related to the societal impact of the COVID-19 pandemic; one topic addressed the impact of the COVID-19 infection itself on the individual.

### Macro Level

#### Pandemic Situation

Some participants talked about aspects that came along with the pandemic situation in general. One aspect was uncertainty in terms of not knowing how the pandemic would develop. Another point is the feeling of isolation. Comments referring to isolation revolved mostly around social isolation due to social distancing. Many participants voiced criticism of the vaccine itself, for example, on its effectiveness. Other criticisms targeted mandatory vaccinations.

I have a feeling of emptiness, seeing my life just floating by and a lot of uncertainty in the future...a lot of loneliness and a strong feeling of not being able to move on because there is no security in the future.

Female, aged 57 years, nonmedical staff; clerk, first study period

#### Government or Politics

Many participants criticized the management of the pandemic by their governments or politicians. Frequently mentioned issues were the lack of trust in politicians and dissatisfaction with political decisions. On the other hand, there were participants who were satisfied with the governmental crisis management:

...Anger at executives for failing to act proactively, particularly over mask distribution and lack of testing. Anger at leaders for conflicting announcements, late, vague pronouncements or actions aimed at getting us back to work at full steam to get the economy back on its feet before the pandemic is quite over.

Female, aged 69 years, nonmedical staff; legal secretary, first study period

#### Social Climate

The participants frequently discussed the social climate. The social climate was indirectly reflected in the comments of many participants, who either directly described it or expressed its effects through comments such as fear, anger, frustration, or annoyance. Many participants talked about a deterioration in emotional well-being unrelated to their working conditions. The unmet needs were mostly discussed during the second study period. Societal split as a social response was directly mentioned by some participants, primarily in relation to the decision to vaccinate. The topic of radicalization was also related to attitudes toward vaccination. Many participants described the phenomenon of questionable information—we call this an “infodemic,” according to the World Health Organization (WHO) [36]—within society, for example, through the media.

People make me sick!

Female, aged 33 years, nonmedical staff; administrator, first study period

I’m so annoyed and can’t hear the word corona anymore.

Female, aged 57 years, nurse, second study period

### Meso Level

#### Measures

Measures in general other than vaccination were frequently discussed by the participants. Topics included the implementation of and reaction to the measures. Participants voiced their complaints about side effects, for example, of wearing masks. There were also comments on the impact of the measures on daily life or work regarding their implementation and inconsistencies.

Wearing the mask for more than 8 hours a day doesn’t exactly help, but we have to live with that for now.

Female, aged 47 years, other job in health care; clinical pharmaceutical laboratory analyst, second study period

#### Working Conditions

Working conditions were a frequently mentioned topic. There were comments on feelings or emotions such as the feeling of pressure, for example, due to high workload and lack of appreciation. Structural changes at work due to the pandemic were mentioned by all profession groups. The HCPs reported on how patient care had changed. Some reported thoughts of quitting their jobs or having already done so. The need for protection and supply was mentioned, for example, in terms of psychological support.

As the *other job in health care* group was very heterogeneous, there were also comments on nursing experiences given by this group. Participants from all professions reported how well or poorly they felt they had been cared for by their employers.

The pressure on the nursing staff has increased greatly as a result of the pandemic and there is little support from politicians and employers.

Male, aged 40 years, nurse, second study period

I miss citizen support and acceptance. It cannot be that nursing staff is denied access to grocery stores.

Female, aged 56 years, nurse, first study period

Recognizing of caregivers only at the peak of the pandemic, and then when the pandemic ends, it is over.

Female, aged 52 years, nurse, second study period

### Micro Level

#### Infection Effects

The experiences of participants who had a COVID-19 infection or experiences about the infection of acquaintances or relatives also emerged, including the long-term effects of a past infection.

To date I have been diagnosed with long COVID. It’s been a year now since I was infected. I still have after-effects [loss of taste and smell, shortness of breath, cough, fatigue, joint pain].

Female, aged 46 years, nurse, second study period

#### Daily Life

Many participants wrote about the upcoming challenges in their daily lives due to the COVID-19 pandemic, mentioning caring for children at home, dealing with vaccine opponents in everyday life and reduced social life in general. However, positive aspects, such as an increase in family activities, were also reported.

I’m in a privileged situation, have a good family life, a big house with lots of outdoor space...

Female, aged 52 years, dentist, first study period

#### Coping

Some participants reported on how they tried to deal with the pandemic. Specifically, exercising and positive thinking seemed to have helped the participants.

In terms of positive feelings, I have been able to adapt by maintaining a healthy lifestyle and exercising at home, taking time to read and after a few weeks appreciating a slower pace of life [before I was very frantic and out of the house all day], I have not felt any fear of contracting the disease, nor have I become obsessed with the subject [I have followed recommended prevention guidelines].

Female, aged 34 years, nonmedical staff; occupational therapist, first study period

### First Versus Second Period

Supporting quotes for the 2 periods are available in Multimedia Appendix 2. For the first study period, participants talked about isolation due to social distancing and voiced a lack of support and protection, for example, regarding PPE. The sense of uncertainty and insecurity was evident in many comments, but there were also comments on the advantages of curfews. During the second study period, vaccination and its effect on society were frequently mentioned topics. The implementation and impact of the COVID-19 pandemic measures were also discussed. Working conditions, measures, social climate, and feelings and emotions were mentioned during both the first and second study periods. All second-level and third-level themes that emerged in the data of the first study period could also be found in the second study period, but some of the second-level themes came up only in the second phase, for example, vaccination and splitting and radicalization of the society. In addition, unmet psychological needs mostly emerged during the second study period.

I don’t feel adequately protected by the protective clothing that we currently have and I am afraid of the day when we will no longer have any protective clothing.

Female, nurse, aged 27 years, first study period

OP's started again...letting everything go back to normal during this time means more work for the same thing.

Female, aged 30 years, nurse, second period

## Discussion

### Principal Findings

In this study, we investigated work-related stressors for physicians and nurses and the coping strategies of both HCPs and nonmedical staff at 2 different periods during the COVID-19 pandemic. In addition, qualitative data were analyzed to gain more information on psychosocial stress factors and unmet needs at different levels of experience of the population during the COVID-19 pandemic crisis.

On the quantitative scale, the uncertainty about when the pandemic will end and the fear of infecting the family were the worst stressors for physicians and nurses at both periods. The most frequent coping strategies for the entire study population were the use of protective measures and active acquisition of knowledge about COVID-19. Significant mean differences were found for stressors such as *Worry about nosocomial spread* (t_752.58_=10.09, *P*<.001) and coping strategies such as *Video-chatting with family and friends by phone to share concerns and support* (t_1218.57_=11.14, *P*<.001).

On the qualitative level, at the beginning of the pandemic, the feeling of insecurity and uncertainty was present, and participants talked about protective equipment and how to handle the social distancing situation. During the second study period, unmet psychosocial needs, exhaustion, and vaccination were frequently mentioned. In the second phase, the topics were more widely spread, as many participants used the free-text fields to “process” their experiences in the previous years.

Overall, these results provide a deeper insight into the perceived problems, burdens, and challenges that our participants faced during the pandemic. By sorting the themes to different levels of experience, the identification of targets for possible necessary psychosocial support was easier. It turned out that the need for psychosocial interventions existed and was even possible to implement at the macro level (eg, more governmental support), at the meso level (eg, working conditions and support of employers), and at the micro level (eg, psychological support).

### Changing of Themes Over the Study Periods

Analyzing the themes over 2 study periods, a shift in topics within the population was observed. Social climate, measures, emotions, and working conditions were frequently discussed during both study periods, but there were different focuses in some places. When the pandemic situation was new to the people and protective gear was in short supply [14], the themes of social distancing reflected feelings of isolation and a general feeling of insecurity and uncertainty. In the second stage, the sentiments of exhaustion and annoyance were more prominent. Vaccination, in general, was a frequently discussed topic in the second period, which in some cases can be connected to comments on the increasing splitting and radicalization of society. An earlier longitudinal study on the challenges of the pandemic found a kind of indignation among their participants in the later stages of the study, whereas at the beginning, participants tended to express a sense of tiredness and monotony [37].

Comments from the second study period mentioned the consequences of the pandemic, such as side effects of the measures or their implementation. Apart from vaccinations, unmet needs, such as additional psychosocial support, mostly emerged in the second study period. A possible explanation might be that in the first study period, the whole situation was new and unknown, so people were busier trying to handle the situation rather than thinking about what could be helpful to them.

This shift in topics illustrates the usefulness of studies at different periods when attempting to understand the psychosocial situation of society during the course of a pandemic. People were overwhelmed by the situation and were more concerned with basic care issues at the beginning, whereas psychosocial burdens and needs seemed to emerge later in time.

### Work Stressors of HCPs and Working Conditions of All Participants

Uncertainty about control over the pandemic and possible infection of family members was the most relevant stressor on a quantitative level for physicians and nurses during both study periods. For the first period, the third and fourth most relevant stressors were concerns about nosocomial spread and constant change in infection control procedures. The measures and their implementation were also discussed in the qualitative comments, especially in the second study period. Considering how often measures, guidelines, and policies have changed [38-40] during the COVD-19 pandemic following the rising or falling infection rates, this concern is easy to understand. Several federal states within a country with differing policies have made this even more complicated [2]. The stressors *Worry about lack of proper knowledge and equipment* and *Worry about nosocomial spread* seemed to be less important for the participants in the second study period, as in the first study period, PPE was in short supply [14].

Working conditions were frequently mentioned in the comments, emphasizing the importance of this theme to participants. As the *other job in health care* group was very heterogeneous, this group also commented on their nursing experiences. One theme was the lack of appreciation, which was frequently mentioned by nursing people. Appreciation at work was proven to be especially important when there were interruptions at work [41], which might be the case in terms of the COVID-19 pandemic. An increased workload, as well as a lack of support from employers, was reported. Feelings of exhaustion, pressure, and thoughts about quitting the job were mostly reported by HCPs. An increase in workload due to the COVID-19 pandemic for HCPs has already been discussed [14], as well as the decline in mental health [17]. Some participants reported how caring for patients has changed or how work processes, in general, have changed due to the pandemic. In addition, telehealth and telework were discussed as working from home increased, especially during the first period of the pandemic [9]. Working from home can indicate a decrease in mental well-being status [42], and it also seemed to be a challenge for some participants in our study. Another European study reported that there were more people who wanted to work from home from the end of 2020 to the beginning of 2021 [9]. However, at this point, the opinions of our participants differed, as many of them also expressed criticism of telemedicine or teleworking regarding the irreplaceability of personal contacts. Staff shortages and high workloads have been a longstanding problem in nursing [15], and there was even more workload during the pandemic [14], as it was also reflected in our study. The fear of staff shortages was also represented on a quantitative level as the third most frequent stressor in the second study period. This feeling might be aggravated by the high COVID-19 infection rates of HCPs in general [3], which worsened again during the omicron wave at the beginning of 2022 [43]. As nurses have been identified as a vulnerable group regarding mental health during the pandemic [17], our results indicated that this professional group needs to be heard for its unmet psychological support.

Vaccination in general and mandatory vaccinations for HCPs was a huge topic in the second study period. This was associated with feelings of being forced and having limited freedom. Regarding working conditions, the emerging themes in our study represent the areas where better preparedness is necessary, for example, proper support by employers, reliable information, employee protection, for example, in the sense of PPE but also regarding workload and work processes, appreciation, more options to combine family and career, and proper psychological support. A European study on working conditions reported a decrease in mental well-being, a decrease in work-life balance for young parents, an increase in people in a financially fragile situation, tiredness with regard to home schooling, and job losses due to the pandemic [9].

### Coping Strategies

When considering the results of the questionnaire on coping strategies, low Cronbach α, which is below the threshold of acceptance, must be considered. The most frequently rated stressors were reflected in the most frequently rated coping strategies. The best coping strategies for the entire study population were to protect themselves and gather more information about the virus. By comparing quantitative data with qualitative comments on coping, the importance of digital offers became clear. The third most often used coping strategy in the first study period was *Video-chatting with family and friends by phone to share concerns and support*, and in the second study period, it was *Engaging in recreational activities (web-based shopping, social media, internet surfing...).* The use of video-chatting may have decreased during the pandemic, as we found a significant decrease in the mean values of the first and second study periods. One reason for this could be the possibility of returning to personal contact during the pandemic. However, it should be noted that few participants also emphasized the non-substitutability of personal contacts by digital contact. On a qualitative level, some participants shared how they dealt with the pandemic situation, for example, by going out, trying to relax, doing hobbies on the web, doing sports, positive thinking, or being with the family. Gaining information was one of the most often used coping strategy on a quantitative level and not being sufficiently informed was also often voiced by participants on a qualitative level, which might be another important approach. Clear information is important for mental health, especially for HCPs [44]. In this context, vocational and educational training for pandemic preparedness of HCPs might be a helpful approach that has already been initiated by various organizations such as the WHO [45].

### Psychosocial Approaches for Future Pandemic Preparedness

Our results emphasize the necessity of an improvement regarding *pandemic preparedness*, which is defined by the WHO as having plans and resources in place to actively respond to a pandemic. It includes, for example, prevention, detection, and containment measures but also plans to respond to any shortages that may arise. Preparing for possible future crises beforehand using the lessons learned from the current pandemic is necessary to face future challenges [46]. In this study, some participants wished for more psychological support. One participant reported that no mental or relaxing therapies were offered at work, whereas another voiced that they were not supported on a psychological level. The stated needs are in line with findings from another study on the psychosocial impact of the COVID-19 pandemic, where the authors argued that support services for future pandemics should be implemented [47].

Previous studies have reported similar needs for HCPs, calling for mental health support services for HCPs in particular [17]. A participant in our study mentioned video- or web-based interventions to help HCPs dealing with stress. The emerging use of e-mental health might be one way to address these psychosocial needs in future crises, as the effectiveness of e-mental health interventions has already been proven [48], and the pandemic has accelerated further advancements [49]. However, it should be noted that few participants also emphasized the non-substitutability of personal contacts by digital contact. Regarding the need for sufficient information, vocational and educational training may be an important approach for future pandemic preparedness. *Infodemic* can have adverse side effects [36] and clear and rapid information for the general population [46] and for HCPs [44], in particular, is important. For future psychometric approaches, attention to the different macro, meso, and micro levels is relevant when collecting psychosocial quantitative data, which might be realized by the combination of qualitative and quantitative approaches.

### Limitations

Several limitations of this study should be considered when interpreting these findings. First, as the questions in our study questionnaire focused on the negative aspects or problems of the pandemic and the open-ended question was very unspecific, the open comments at the end of the study may also be biased toward negative statements or inappropriate statements. Second, the link to the web-based survey was distributed via social media and via the personal and professional networks of the authors. As the contact networks in the individual European countries were not equally strong and web-based distribution was difficult to control, the number of participants for each country was different. Despite the large sample size, these results cannot be generalized for Europe as our participants came from only 10 countries out of the 27 European Union countries. Furthermore, only a small subset of the entire population provided qualitative feedback. Third, the different groups within the HCP group of nurses, physicians, and other professionals in health care organizations were somewhat heterogeneous, as well as the group of nonmedical staff, of whom not all revealed their actual profession. We have attempted to explain these groups in as much detail as possible.

Moreover, the study phases did not always occur at the exact time of the lockdowns or peaks in infection rates in individual European countries, which may have influenced the results. At best, there was a partial overlap between respondents in the first and second study periods. The validation of questionnaires on stressors and coping should be part of future studies.

### Conclusions

This mixed methods study provides valuable insights into the individual and psychosocial problems faced by European HCPs as well as nonmedical staff over the course of 3 years of the COVID-19 pandemic. Open-ended questions supplemented our quantitative surveys to identify the core problems on different levels of experience of the individuals. Using thematic analysis of qualitative data, we identified 8 major themes of experiences in the pandemic situation: *government or politics*, *social climate*, *measures*, *working conditions*, *infection effects*, *daily life*, and *coping mechanisms*. These different levels of experiences could be assigned to the macro, meso, and micro levels of social structures.

Uncertainty about the end of the pandemic and the fear of infecting family members were identified as the most important stress factors for physicians and nurses. The most commonly used coping strategies of the entire study population were the use of protective measures and acquiring up-to-date information on the COVID-19 pandemic. In the first study period, social distancing, uncertainty, and the need for protection were the topics mentioned. During the second study period, topics such as vaccination, exhaustion, and unmet psychosocial needs emerged. Working conditions were frequently discussed by all participants during both periods. An improvement in pandemic preparedness, with emphasis on vulnerable groups such as HCP in general and nurses in particular, is needed. Several psychosocial approaches should be considered for future research, for example, the development of easily accessible digital psychosocial services and educational and vocational training.

## Appendix

Multimedia Appendix 1 Description of the themes with supporting quotes.

Multimedia Appendix 2 Supporting quotes for the first versus second study period.

## Abbreviations

HCP: health care professional
ICU: intensive care unit
PPE: personal protective equipment
SARS: severe acute respiratory syndrome
WHO: World Health Organization

## Data Availability

The data sets generated during and analyzed during this study are available from the corresponding author upon reasonable request.
