# Supplementary material for: Unmet Psychosocial Needs of Health Care Professionals in Europe During the COVID-19 Pandemic: Mixed Methods Approach
Source: JMIR Public Health Surveill. 2023 Sep 6;9:e45664. doi: 10.2196/45664 (PMC10484324; doi:10.2196/45664)
Supplement: Multimedia Appendix 1 [file publichealth_v9i1e45664_app1.docx]

| **Level** | **First-level themes** | **Definition** | **Second-level themes** | **Definition** | **Third-level themes** | **Supporting quotes** |
| --- | --- | --- | --- | --- | --- | --- |
| Macro | Pandemic situation | includes all comments on the new living situations caused by the pandemic situation. | Uncertainty | The state of not knowing how the pandemic situation will develop |  | *The uncertainty about what will happen next is what bothers me the most*  (female, 26, nurse, 1^st^ study period) |
|  |  |  | Standstill | Feeling of a state without activity, development |  | *Feeling that projects are not progressing* (female, 43, other job in health care system (health care manager), 2^nd^ study period) |
|  |  |  | Isolation/  Loneliness | The feeling of being more and more alone and separated from other people |  | *At the beginning of 2020 isolation hit me extremely. The distance from my children was unbearable.* (female, 54, nurse, 2^nd^ study period*)* |
|  |  |  | Vaccination | General statements about vaccination or its negative effects/aspects. | Criticism | *Too much pressure, according to the law we cannot force the society to be vaccinated, the vaccine is still not on the market, a vaccination that you do every 6 months is no longer a vaccination but a therapy, discrimination, violation of freedom* (female, 39, nurse, 2^nd^ study period) |
|  |  |  |  |  | Effects | *The booster made me very ill, so last week in a very bad mood, always sad, left alone, confusing measures*  (female, 42, nurse, 2^nd^ study period) |
|  | Government or Politics | Positive and negative mentioned aspects regarding politics and/or government |  |  |  | *You feel abandoned by the government and not taken seriously (obligation to go to work despite severe illness or positive test results)*  (female, 26, nurse, 1^st^ study period) |
|  | Social  climate | Includes all comments that describe the current interpersonal situation or how people interact or communicate with each other. | Affects | Emotional arousal |  | *There are so many people who are scared at the moment about the pandemic, which is then constantly being maintained through the media and politics.*  (female, 54, nurse, 2^nd^ study period) |
|  |  |  | Post covid life | Thoughts/hopes about life after the pandemic |  | *It's time for normality to come back and I want to go on a normal vacation without masks and Covid.*  (female, 56, nurse, 2^nd^ study period) |
|  |  |  | Emotional well-being | General comments regarding emotions and feelings not related to work but related to the pandemic situation in general |  | *The pandemic exhausted my resilience reserves last year.*  (male, 26, other job in health care (medical student in his practical year), 2^nd^ study period) |
|  |  |  | Unmet needs | General comments regarding need for telemedicine or other needs that weren´t met, unmet needs at work are not included |  | *Everyone should benefit from psychological aftercare and relaxation opportunities due to the duration of the pandemic.*  (female, 47, other job in health care (unknown), 2^nd^ study period) |
|  |  |  | Social response | Reactions to the pandemic within society | Splitting | *The division of society worries me.*  (female, 39, doctor, 2^nd^ study period) |
|  |  |  |  |  | Radicalization | *Great concern about civil war-like conditions, since vaccination refusers, lateral thinkers, etc. are becoming more and more aggressive.*  (male, 42, other job in health care (educator), 2^nd^ study period) |
|  |  |  |  |  | Incomprehension | *In me, the lack of understanding and the aggression against vaccination refusers and corona deniers has increased noticeably in the last few weeks.*  (male, 33, other job in health care (anesthesia nurse), 2^nd^ study period) |
|  |  |  |  |  | Support | *It is very important to me to take care of my professional group (care!) and to support them!*  (female, 57, other job in health care (quality management representative, patient contact in a gerontopsychiatric department), 2^nd^ study period) |
|  |  |  | Infodemic | According to the definition of the WHO [36], all comments are included regarding too much, false or misleading information about the pandemic in the media ore by other sources like employer |  | *The growing disinformation is a particular challenge and its acceptance is more worrying than COVID*  (female, 50, other job in health care (midwife), 2^nd^ study period) |
| Meso | Measures | At work and in daily life (problems with masks etc.) Includes all comments on problems with masks, testing, etc. But also general statements about burdens experienced through the measures. | Side effects | Consequences of the measures that are mostly undesirable |  | *Wearing a mask is the hardest thing for me to endure. It's a serious barrier to communication, especially in our field of , psychiatry.*  (male, 39, nurse, 2^nd^ study period) |
|  |  |  | Implementation criticism | Comments on the way the measures were integrated into everyday life or work |  | *The processes of the offices for PCR testing and quarantine are in need of improvement*  *(female, 42, non-medical staff (student/intern),* 2^nd^ study period*)* |
|  |  |  | Inconsistencies | Contradictions and volatility regarding measure implementation |  | *(...) Equally stressful are frequent changes in corona regulations and rules, both at work and in private life.*  (female, 57, nurse, 2^nd^ study period) |
|  |  |  | Response | Social movements or reactions to adapt to the measures |  | "I dealt early and intensively with online coaching and also created outdoor offers. This way, practically all appointments could take place - no matter where and how!*!*  (female, 57, other job in health care (quality management representative, patient contact in a gerontopsychiatric department), 2^nd^ study period) |
|  |  |  | Desire of  freedom | When comments address the wish to live without measures again |  | *I can’t take the restrictions anymore, I need my freedom back!!!!!!!*  (female, 45, other job in health care (kinesitherapist/ physiotherapist), 2^nd^ study period) |
|  | Working conditions | All mentioned aspects that relate to negative experiences at work due to the pandemic situation | Employer | All comments that talk about negative experiences with employers |  | *I felt a great lack of consideration and respect from medical professionals and management"* (female, 55, non-medical staff (higher technician), 1^st^ study period) |
|  |  |  | Lack of appreciation/  support | When employees talk about not feeling appreciated for their work during pandemic or not feeling supported. |  | *The hypocrisy of suddenly being systemically relevant only to be forgotten again later makes me angry*  (female, 54, nurse, 1^st^ study period) |
|  |  |  | Need for protection | When employees talk about the protection/lack of protection or need for protection from the virus at work. |  | *I don't feel adequately protected by the protective clothing that we currently have and I am afraid of the day when we will no longer have any protective clothing*  (female, 27, nurse, 1^st^ study period) |
|  |  |  | Feelings/  Emotions | When participants talk about their feelings or emotions (stressed, tired, burdened...) that they have regarding their work during the COVID pandemic |  | *I often regret my decision to work in nursing*  (male, 32, nurse, 2^nd^ study period) |
|  |  |  | Mental health | Comments on symptoms of mental illnesses like depression, anxiety disorder, sleep disorder |  | *At the end of 2020, I was hospitalized for somatic stress disorder following arbitrary changes in my services from my employer. In total, I was unable to work for more than three months*  (male, 45, other job in health care (quality management representative), 2^nd^ study period) |
|  |  |  | Compatibility of family and career | Comments that address aspects of combining family and work during the pandemic |  | *Nursing couple with 1 baby of 17 months. Both working. Reconciling shifts so that one of us can always be with him at home. These times have been tough physically and emotionally.* (female, 32, nurse, 1^st^ study period) |
|  |  |  | Workload | Comments regarding the amount of work during the pandemic |  | *Big overload of daily work. I feel on the verge of physical and mental exhaustion.*  (male, 49, non-medical staff (communication), 2^nd^ study period) |
|  |  |  | Supply | Everything regarding medical/non medical needs of the patients/employees |  | *I noticed (and that's just my opinion and how I feel about the situation). I think that we were not looked after on a psychological level. I think this is an important point that deserves attention.*  (female, 30, nurse, 2^nd^ study period) |
|  |  |  | Structural limitations | Work and vaccination  Changed processes (surgeries, holiday bans...)  Holidays  Staff shortage  No masks/protective equipment  general criticism of the health care system |  | *Great concern about the lack of protective clothing, FFb masks, etc. for carers and doctors*  (male, 68, doctor, 1^st^ study period) |
| Micro | Infection effects | All statements describing in general the course/side effects etc. of an infection with COVID-19 together. |  |  |  | *To date I have been diagnosed with long COVID. It's been a year now since I was infected. I still have side-effects (loss of taste and smell, shortness of breath, cough, fatigue, joint pain).*  (female, 46, nurse, 2^nd^ study period) |
|  | Daily life | All statements that focus on changes/challenges in everyday life due to the pandemic. |  |  |  | *We have a multi-generational house (currently no direct contact with the grandparents) Exactly those two are my biggest stress factor because they put the pandemic into perspective. Also, I feel like I'm being watched all the time. I would like to move out*  (female, 39, nurse, 1^st^ study period) |
|  | Coping | This includes all comments that deal with ways of coping or dealing with the situation. |  |  |  | *A feeling of loneliness, I go for a run and given this pandemic situation, it helps me a lot. Mentally.*  (female, 45, other job in health care (medical information technician), 2^nd^ study period) |
|  | Survey | Comments on the questionnaire or the survey itself. | Critics | Aspects of the survey that could be improved or which the participants did not like |  | *Strange that there was no before and after comparison so far, as people can also find it quite comfortable to work in the home office and/or to live a little more "isolated". The premise of this study is obviously that "everything" associated with the CoViD19 pandemic <must> be bad?!*  (female, 35, other job in health care (research assistant (health services research), 2^nd^ study period) |
|  |  |  | Additional information | If participants wanted to share any additional information related to the survey questions |  | *When asked about my home office: I sometimes work at home, sometimes I see clients; namely those who cannot be expected to remain alone*  (female, 49, non-medical staff (mobile caregiver), 1^st^ study period) |
|  |  |  | Thankfulness | All comments expressing gratitude for the study |  | *I find this study very interesting and I thank you for it.*  (female, 46, other job in health care (masseur kinesitherapist/ physiotherapist), 1^st^ study period) |
