# Supplementary material for: Unmet Psychosocial Needs of Health Care Professionals in Europe During the COVID-19 Pandemic: Mixed Methods Approach
Source: JMIR Public Health Surveill. 2023 Sep 6;9:e45664. doi: 10.2196/45664 (PMC10484324; doi:10.2196/45664)
Supplement: Multimedia Appendix 2 [file publichealth_v9i1e45664_app2.docx]

| **Level** | **First-level themes** | **First study period** | **Second study period** |
| --- | --- | --- | --- |
| Macro | Pandemic situation | *(…)the most difficult thing about this whole period is the fear for the people we love and not being able to see them and kiss them and not knowing when I will be able to do that. I haven't seen my children for 30 days (…).* (Female, 41, doctor,) | *We're just living in a permanent state/loop of waiting and seeing at the moment. This is very tiring.*  (female, 44, nurse) |
|  |  |  | *I am triple vaccinated and followed all the measures taken by the governments. I'm feeling worse and worse about this pandemic. I don't think we'll ever get away from it, and that scares me (…).* (female, 52, other job in healthcare (care assistant))) |
|  |  |  | *Teleworking and exclusive video contact have destroyed human relationships (work and personal).* (female, 63, non-medical staff, (Clinical Research and Innovation Department (DRCI))) |
|  | Government or politics | *(…) Anger at executives for failing to act proactively, particularly over mask distribution and lack of testing. Anger at leaders for conflicting announcements, late, vague pronouncements or actions aimed at getting us back to work at full steam to get the economy back on its feet before the pandemic is quite over*. (Female, 69, Non-medical staff (legal secretary),) | *The country has done a good job so that staff can be tested regularly, for protective measures, for vaccinations within the country.* (female, 55, nurse) |
|  |  | *I felt that this period deeply affected my emotions and "well-being", although I am a positive and constructive person, the feeling of being unable to do anything to protect myself, compounded by the disastrous management of our leaders (FRANCE), was pretty hard to take. Thank you for your study, it is obvious that the psychological aspect of this period must be taken into account. It is a necessity.*  (male, non-medical staff (carpenter), 46) | *Currently I have the feeling that the federal government has not decided on enough restrictions, as if nothing was learned from the previous waves. Many measures will (if at all) come too late and push the health system to its limits. I look to the future with a very bad feeling due to the spread of the Omicron variant.*  (female, 33, non-medical staff (administrator)) |
|  | Social  climate | *My extreme concern is related to my illness and my financial and social insecurity rather than the virus itself.*  (female, non-medical staff (translator), 37) | *I'm so annoyed and can't hear the word Corona anymore.* (female, 57, nurse) |
|  |  | *People make me sick!* (female, 33, Non medical (administrator),) | *Stop discriminating between the vaccinated and the unvaccinated*  (male, non-medical staff (systems engineer), 48) |
|  |  | *The feeling of being left alone with the situation and not knowing what is really true.*  (male, non-medical staff (teacher), 59) |  |
| Meso | Measures |  | *Wearing the mask for more than 8 hours a day doesn't exactly help, but we have to live with that for now.* (female, 47,other job in healthcare (clinical pharmaceutical laboratory analyst))  ) |
|  |  | *I don't feel adequately protected by the protective clothing that we currently have and I am afraid of the day when we will no longer have any protective clothing.*  (female, nurse, 27) | *„I dealt early and intensively with online coaching and also created outdoor offers. This way, practically all appointments could take place - no matter where and how!"* (female, 57, other job in health care (quality management representative, patient contact in a gerontopsychiatric department)) |
|  |  | *The lockdown has been a beneficial experience for my married life and my family life…*  (male, non-medical staff (manager), 40) |  |
|  |  | *The curfew was pure happiness for me!*  (male, non-medical staff (administrator) 33) | *OP's started again... letting everything go back to normal during this time means more work for the same thing.* (female, 30, nurse) |
|  | Working conditions | *I miss citizen support and acceptance. It cannot be that nursing staff is denied access to grocery stores.* (Female, 56,nurse) | *almost no quality of work the organization is almost nonexistent*  (female, 22,non-medical staff (secretary radiology) |
|  |  | *There is almost no protective equipment in my facility, and there is no emergency plan.* (female, 26, nurse) | *Big overload of daily work. I feel on the verge of physical and mental exhaustion.*  (male, 49, non-medical staff (communication)) |
|  |  |  | *The pressure on the nursing staff has increased greatly as a result of the pandemic and there is little support from politicians and employers*. (male, 40, nurse) |
|  |  |  | *"I am not vaccinated and since Saturday a law has been passed and I feel obliged to be vaccinated as if I were held hostage because if I am not vaccinated I will no longer have the right to work and I will not be paid."?*(female, 47, nurse) |
|  |  |  | *I can no longer take care of Covid patients let alone unvaccinated patients. My empathy is no longer present with unvaccinated patients.* (female, 30, nurse) |
| Micro | Infection effects | *My neighbour has died from Covid-19*  *My Counsellor has died from Covid-19*  *Both were over 80 years*  (female, non-medical staff (retired), 66) | *I have been affected by Covid long.*  (female, 26, non-medical staff (psychologist)) |
|  | Daily life | *I'm less afraid of infecting myself but much more that one of my family members will be infected*  (female, nurse, 27) | *As parents, everyday life is extremely difficult, no family help because it is risky and emotionally overloaded (test without a test, school without school, teleworking not always possible with three children and two sick ones, lack of understanding of the supervisors“).*  (female, 41, non-medical staff (office)) |
|  | Coping | *I have been able to continue with some of my hobbies by doing them online*  (male, non-medical staff (retired), 69) | *I can only speak for myself, you can also gain positive things from every crisis, we did more handcrafts and played at home again, we have a dog that we walked a lot withand when we could, we also took part in activities. Think positive and life goes on.*  (female, 55, non-medical staff (administration)) |
